# Measurement of Water Absorption of Recycled Aggregate

**DOI:** 10.3390/ma15155141

**Published:** 2022-07-25

**Authors:** Zhenhua Duan, Wenjing Zhao, Taohua Ye, Yunhui Zhang, Chuanchuan Zhang

**Affiliations:** 1 Department of Structural Engineering, College of Civil Engineering, Tongji University, Shanghai 200092, China; zhduan@tongji.edu.cn (Z.D.); 1650818@tongji.edu.cn (W.Z.); 2 College of Environmental Science and Engineering, Tongji University, Shanghai 200092, China; yunhuizhang@tongji.edu.cn; 3 Shanxi Transportation Technology Research and Development Co., Ltd., Taiyuan 030000, China; zccmech2012@163.com

**Keywords:** recycled aggregate (RA), water absorption (W_a_), saturated surface-dry (SSD), measurement

## Abstract

Owing to the high absorption capacity of recycled aggregate (RA), it is crucial to accurately measure its saturated-surface-dried water absorption (W_SSD_), which largely affects an effective water-to-binder ratio of recycled aggregate concrete. In this study, existing measurement methods for the W_SSD_ of RA are extensively reviewed, including *Wiping*, *Slumping*, *Centrifugation*, *Infrared*, *Evaporation*, *Airflow drying*, *Conductivity*, *Pycnometer*, *Hydrostatic balance*, and *Extrapolation*. In particular, the physical principles and operability of these methods are emphasized. It was determined that the accuracy of all test results was not satisfactory. For example, the water in pores with an open-ended direction that was opposite to the centrifugal force could largely be retained. In *Airflow drying*, the temperature change was significantly delayed. In addition, in *Hydrostatic balance*, RA would pre-absorb water before determining the initial reading. Therefore, several suggestions for optimizing these methods are presented, such as the combination of *Evaporation* and *Airflow drying*, the liquid selection in *Hydrostatic balance*, and the addition of a tiny mixer in each centrifuge tube. In summary, this review facilitates the development of an accurate and convenient method for measuring the W_SSD_ of RA.

## 1. Introduction

In China, the exploitation of natural aggregate (NA), especially river sand, has caused serious environmental problems [1,2]. For example, because of the overexploitation of river sand, the ecological environment of Poyang Lake (the largest freshwater lake in China) has been severely eroded [3,4]. However, the development rate of the construction sector has not slowed down. In 2021, the total demand for aggregates in China set a new record of 17.89 billion tons [5]. Therefore, it is crucial to overcome the out-of-balance relationship between urgent environmental protection and huge construction demand. A promising method is to prepare recycled aggregate concrete (RAC) using recycled aggregate (RA) from construction and demolition (C&D) waste. As a green building material, the raw materials of RAC have various sources and abundant reserves [6,7,8]. In 2021, more than 1.8 billion tons of C&D waste were discarded in China, where waste concrete and brick accounted for more than 80% [9]. Currently, the properties of RA and RAC have attracted huge attention [10,11,12].

In general, RA can be prepared by crushing waste concrete and/or brick, of which the main difference from NA is the presence of attached old mortar (OM) that leads to higher water absorption (W_a_) of RA [13,14,15]. Therefore, the preparation procedures of natural aggregate concrete (NAC) are different to those of RAC. More specifically, the latter needs to consider the absorption of RA in the slurry, which is usually calculated by its saturated-surface-dried water absorption (W_SSD_) [16]. Clearly, the accurate evaluation of the W_SSD_ of RA is of great significance to control an effective water-to-binder (w/b) ratio (E_w/b_) of RAC [17]. When the measured W_SSD_ is not accurate, the E_w/b_ would deviate from the calculated value and further affect the properties of RAC. For example, a higher E_w/b_ may cause the slurry to bleed [18], while the slurry with a lower E_w/b_ may not meet the requirements of workability [19]. Furthermore, a higher E_w/b_ would degrade the strength of RAC [20]. The accurate evaluation of W_SSD_ also affects other properties of RAC, such as alkali-silica reaction [21] and creep [22]. Currently, several methods have been proposed to measure the W_SSD_ of RA, including *Wiping*, *Slumping*, *Centrifugation*, *Infrared*, *Evaporation*, *Airflow drying*, *Conductivity*, *Pycnometer*, *Hydrostatic balance*, and *Extrapolation*.

In our previous study [23], *Slumping* was used to measure the W_SSD_ of RA. It was found that the results of the same batch of samples obtained by the same operation were quite different due to the vague definition of the slumped shapes. In another previous study [24], *Hydrostatic balance* was applied to measure the W_SSD_ of RA derived from waste concrete and brick. It was found that the W_SSD_ of RA was underestimated owing to its pre-absorption before determining the initial reading. Furthermore, we detected in *Evaporation* that when the inflection point of the curve representing the W_SSD_ of RA appeared, the sample at the edge of the dry plane was under the state of air dry [1]. In addition to the above methods, *Centrifugation* was also used to measure the W_SSD_ of RA derived from waste concrete and brick [25]. Based on centrifugal parameters proposed by Miller et al. [26], a single aggregate with a wet side and a dry side was frequently detected. Obviously, because they depend on different physical principles and operation procedures, there are differences in the test accuracy of the above methods. However, even so, the overview of these methods has not yet been reported.

Therefore, this paper reviews all the measurement methods for the W_SSD_ of RA for the first time. In particular, the physical principles and operability of these methods are emphasized. In addition, water absorption behaviors and the moisture states of RA are discussed in detail. Finally, several suggestions to optimize existing methods for measuring W_SSD_ are provided. In summary, this paper facilitates the development of an accurate and convenient method for measuring the W_SSD_ of RA.

## 2. Absorption Behaviors and Moisture States of RA

### 2.1. Absorption Behaviors

**Figure 1:**
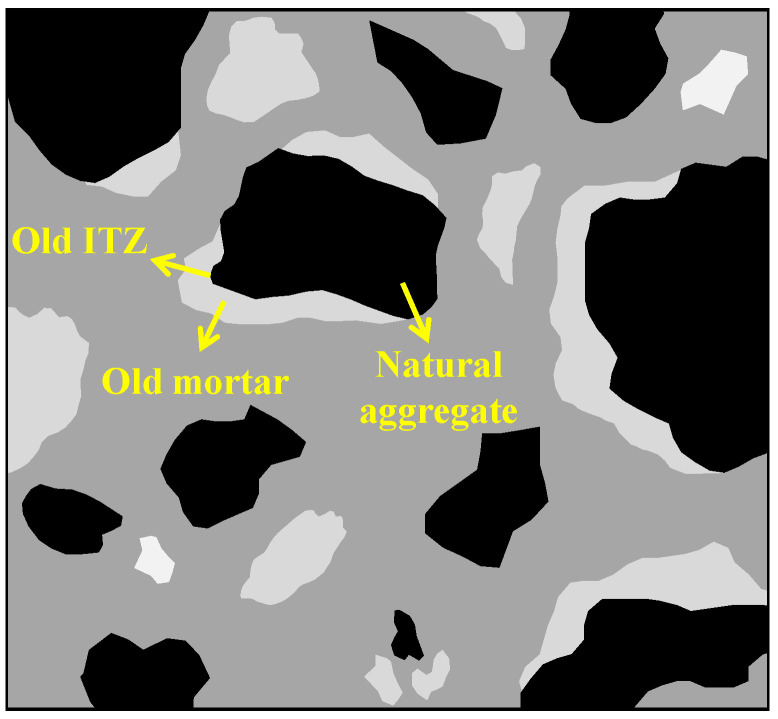
Cross-sectional schematic of RAC.

As shown in Figure 1, RA usually consists of NA, OM, and an old interfacial transition zone (ITZ) between them [27,28]. Therefore, the absorption behaviors of RA are quite different to those of NA. Firstly, RA has a higher W_a_ than NA. On the one hand, the shrinkage during cement hydration produces a number of capillary pores in OM [29,30]. On the other hand, hydration products interlace to form a spatial network structure, generating many inter-hydrate pores in OM [31,32]. In addition, during the preparation of RA, microcracks would be formed in OM due to the impact load of a crusher [33,34]. Consequently, these defects provide a lot of channels for water transport.

Secondly, the W_a_ of RA has a larger standard deviation than NA, which is mainly attributed to the properties of parent concrete (PC) [35,36]. In general, PC-related factors such as mixing proportions (e.g., cement type [37] and water content [38]), designed strength [39], service life period [40], and exposure environment [41] have a significant impact on the quality and content of OM. As a result, different RAs prepared from different PCs have huge differences in the morphology and microstructure of OM.

Thirdly, the W_a_ of RA has an obvious time-varying law with two outstanding characteristics. On the one hand, the absorption rate of RA can reach the peak value in the early seconds [42]. Agrela et al. [43] indicated that the absorption amount of RA in the first 10 min could reach 85% of that at 24 h. Duan et al. [24] further reported that the absorption amount of RA in the first 15 s could reach 46.7% of that at 24 h. On the other hand, the absorption rate of RA in still water can sharply decrease with time [44], as shown in Equation (1).

(1) $$W_{a}=A\times (1-e^{-\mathrm{kt}})+B\times \sqrt{t}$$

where A, B, and k are constants and t is the time.

Finally, with the decrease in particle size, both the absorption amount and the rate of RA would increase significantly. There is good evidence that the W_SSD_ of recycled fine aggregate (RFA) is much larger than that of recycled coarse aggregate (RCA) [45]. This is because RA with a smaller particle size usually has a larger specific surface area and a higher mass ratio of OM.

### 2.2. Moisture States

As described above, the pore characteristics of RA largely determine its W_SSD_. Here, the W_SSD_ usually refers to the W_a_ of RA when open pores are filled with water and the aggregate surface is dry, and the corresponding state is called the saturated-surface-dried state. Furthermore, there are other moisture states according to the location and amount of water in RA. Tegguer et al. [46] divided moisture states of RA into five categories, i.e., oven dry (OD), air dry (AD), surface saturation and surface-dry (SSSD), saturated surface-dry (SSD), and over saturation (OS), as shown in Table 1. The absorption capacity of RA gradually decreases following this order. Note that when characterizing absorption, the behaviors of RA, AD and SSSD states are usually considered to be identical, since their differences are not significant at the macroscopic scale.

Poon et al. [47] prepared RCA at AD, OD, and SSD states and studied the effects of different RCAs on the workability and mechanical properties of RAC. The influence of different moisture states (i.e., AD, OD, and SSD) of RFA and RCA on the properties (e.g., cracking and chloride resistance) of RAC was also investigated [18,24,48]. The results showed that different moisture states of RA have various impacts on the properties of RAC [49]. In particular, the AD state can be regarded as an ideal moisture state for RA, because RA at the AD state can enable fresh RAC with good initial fluidity without excessive loss with time and hardened RAC with better mechanical properties and durability.

However, the above studies did not propose a unified definition of the W_a_ of RA at the AD state. As shown in Table 1, RA at the AD state has a higher absorption capacity than that at the SSD state. Therefore, the common way to prepare air-dried RA in the slurry is described as follows. As shown in Equation (2), the W_SSD_ of RA is firstly calculated and then multiplied by a reduction coefficient to obtain the amount of additional water that can be added to the slurry during the RAC preparation or used to pre-soak RA before the preparation. Another reason for adopting the reduction coefficient is that cement particles would form a water-blocking film on the surface of RA so that the latter cannot ever reach the SSD state inside the slurry [50].

(2) $$M_{\mathrm{aw}}{=M}_{a}{\times \alpha \times W}_{\mathrm{SSD}}$$

where M_a_, M_aw_, and α are the mass of RA, the mass of additional water, and a reduction coefficient, respectively. The value of α has been discussed in detail in a recent review [50]. In general, when the w/b ratio of RAC ranges from 0.35 to 0.50, α should be taken as 72–80% [51]. This is because RAC can obtain excellent workability and superior mechanical properties simultaneously when α is taken as >70% [52].

## 3. Measurement Methods for W_SSD_ of RA

### 3.1. Standard Methods

In general, the measurement for the W_SSD_ of RA can be conducted as follows: (1) placing RA in an oven at 105 ± 5 ℃ for 24 ± 0.5 h to reach the OD state and recording the mass of RA as M_1_; (2) soaking RA in water for 24 h to reach the OS state; and (3) processing RA from the OS state to the SSD state and recording the mass of RA as M_2_. Therefore, the W_SSD_ of RA can be calculated using Equation (3).

(3) $$W_{\mathrm{SSD}}=\frac{M_{2}-M_{1}}{M_{1}}\times 100\%$$

Obviously, step (3) is the key in the above series of steps, because the judgment of whether RA reaches the SSD state directly affects the calculation accuracy of W_SSD_. At present, the measurement methods for W_SSD_ of RCA and RFA in standards include *Wiping* and *Slumping*, respectively. These methods have been widely used in actual projects due to low dependence on equipment, high operability, and its simple calculation.

#### 3.1.1. Wiping

(1) *Wiping* by towel

According to NF EN 1097-6 [53], BS 812: part 2 [54], and ASTM C127-12 [55], RCA at the OS state can be wiped with a dry towel to remove the excess water on its surface and to further make it reach the SSD state. Similarly, GB/T 25177 [56] suggests that RCA can be placed on a towel groove and rolled back and forth to absorb the excess water on its surface.

Obviously, the physical principle of *Wiping* is that excess water would migrate from the surface of RCA to the dry towel due to a moisture gradient. However, *Wiping* has a lot of problems. On the one hand, it is difficult to judge whether RCA reaches the SSD state, since the judgment always depends on an operator’s experience. If the wiping duration is too short, the surface water of RCA may not be completely removed, resulting in larger values of M_2_ and W_SSD_. This is because RCA generally has a rough surface texture along with small pits. If the wiping duration is too long, the towel may suck out a little pore water of RCA, resulting in small values of M_2_ and W_SSD_. On the other hand, the rough surface of the towel may squash the adhered OM. Consequently, a small amount of OM is damaged, causing the values of M_2_ and W_SSD_ to be underestimated.

(2) *Wiping* by tissue

To overcome the above problems, Castro et al. [57] proposed to use tissues instead of towels, referring to NY 703-19 E [58]. Compared with towels, tissues are more absorbent and more deformable. Therefore, tissues can absorb more water, especially in the small pits on the surface of RCA. Moreover, it is easier to judge whether water is absorbed, because the moist tissue would become soft. The French Ministry of Transportation [59] further suggests conducting *Wiping* using colorful absorbent tissues, which enables more effective judgment of whether RCA reaches the SSD state. In addition, the advantage of *Wiping* by tissues also includes less squashed OM, because the surface of the tissue is smoother and its friction effect on the surface of RCA is smaller. However, as shown in Table 2, *Wiping* by both towel and tissue results in the same problem, i.e., the judgment of the SSD state of RCA is always affected by an operator’s experience.

#### 3.1.2. Slumping

**Figure 2:**
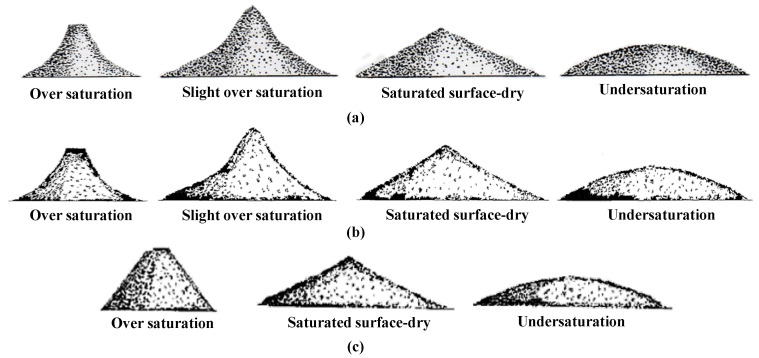
Definitions of the slumped shape of RFA in IRAM 120: 2002 (**a**), EN 1097-6: 2013 (**b**), and GB/T 14684-2011 (**c**).

In most standards, *Slumping* has been widely used to measure the W_SSD_ of RFA. According to GB/T 14684 [60], the details of *Slumping* are described as follows: (1) drying evenly the water on the surface of RFA at the OS state with a hair dryer; (2) filling RFA into a mold two times and further compacting; and (3) lifting the mold and observing the shape of RFA after slumping. The operation process in other standards is similar to the above, as shown in Table 3. However, different standards have different definitions of the slumped shape of RFA at the SSD state, as shown in Figure 2.

Apparently, the accuracy and repeatability of *Slumping* are debatable due to different definitions of the slumped shape of RFA at the SSD state in different standards. For example, in EN 1097-6 [53], the slumped shape of RFA at the SSD state is conical, while in ASTM C128-12 [61], the slumped shape is described as a slight slump on one side. Furthermore, the judgment of the slumped shape of RFA depends on an operator’s experience, which greatly increases the uncertainty of the test results. For instance, for ASTM C128-12 [61], different operators have various understandings of ‘slight slump’.

By comparing *Slumping* with *Wiping*, Kim et al. [63] indicated that with the decrease in the particle size of RFA, the reliability of *Slumping* gradually decreased. More specifically, when the particle size is below 0.60 mm, the results of *Slumping* are much smaller than *Wiping*. It can be attributed to a higher surface roughness of RFA with a smaller particle size. Consequently, although losing the surface tension provided by water, finer RFA still remains stable without any slump under friction force. In addition, finer RFA always has a higher content of impurities, such as cement paste powder and dust, that can absorb water and further agglomerate [64,65]. As a result, although the surface of finer RFA is dry, a lot of water can be left inside. Therefore, during the lifting of the mold, the aggregate is disturbed and the inter-particle water is released, increasing the cohesion between aggregates by surface tension and resulting in a lower W_SSD_.

### 3.2. Centrifugation

Miller et al. [26,66] developed a device that can quickly dry the surface of porous lightweight aggregates by centrifugal force. When the centrifugal force overcomes the adhesion force of the surface water of the aggregates, the surface water would escape from the aggregates. Furthermore, the authors proposed a series of test procedures: (1) placing lightweight aggregates in a centrifuge that can filter water; and (2) operating the device at 2000 rpm for 3 min to remove the surface water of aggregates. Moreover, the authors recruited 25 volunteers to conduct *Centrifugation* following these procedures [67]. It was found that compared with *Wiping*, the outputs of *Centrifugation* were more stable and had a smaller standard deviation, indicating that *Centrifugation* could exclude the interference of human factors to some degree.

Li et al. [68] further applied *Centrifugation* for the W_SSD_ of RFA and established the relationship between a pore radius that could totally empty the pore water and centrifugal parameters (i.e., rotational speed and centrifugal radius), as shown in Equation (4).

(4) $$r=\sqrt{\frac{6\gamma \cos\theta }{{R\rho \omega }^{2}}}$$

where r, R, θ, γ, ρ, and ω are pore radius, centrifugal radius, contact angle, the surface tension of water, water density, and the angular velocity of a centrifuge, respectively. When the temperature is 20 °C, γ and ρ are 72.5 mM/m and 1000 kg/m^3^, respectively. In addition, ω can be calculated by the equation of ω = 2πn, where n is the rotational speed.

**Figure 3:**
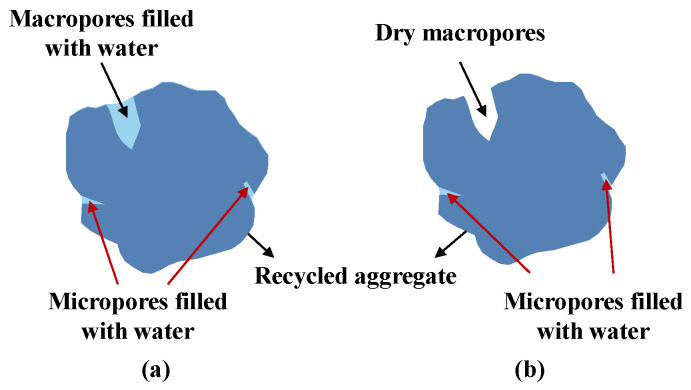
Comparison of old calculation guideline (**a**) and new calculation guideline (**b**) for the W_SSD_ of RA.

According to Kim et al. [69], when calculating the W_SSD_ of RA, the water in macropores of RA should be neglected, as shown in Figure 3. Therefore, the maximum pore radius containing pore water should be specified, which can be realized by controlling the rotation speed of the centrifuge, as shown in Equation (4). Consequently, the water in larger pores can be emptied while the water in smaller pores can be reserved. Based on this theory, Li et al. [68] reported that after centrifuging at 2000 rpm for 10 min, all RFAs with different sources could reach the SSD state.

However, it should be emphasized that pore radius is not the only factor affecting the moisture state of RFA. Since the direction of centrifugal force is fixed during *Centrifugation*, the water of RFA would always migrate in a fixed direction, resulting in the water within pores facing the other direction being largely retained. Furthermore, since the distribution and open-ended direction of pores in OM are random, it is very difficult for *Centrifugation* to create a moisture state where all large pores are dry and all small pores are saturated.

### 3.3. Infrared

**Figure 4:**
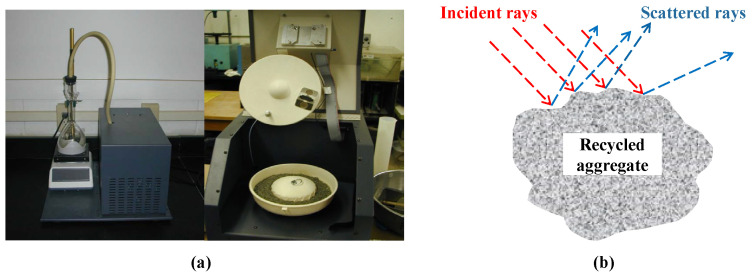
Photos of the SSDetect system (**a**) and reflection of infrared lights (**b**).

To measure the W_SSD_ of fine aggregates, the Thermolyne Company invented a device called the SSDetect system. As shown in Figure 4a, the system consists of an orbital mixer, a calibrated water injection pump, an infrared source, an infrared detector, and a mixing bowl [70]. In the beginning, the system would continuously spray water on the aggregates in the bowl and further stir them. Because of the irregular surface of the aggregates, the incident infrared rays would be reflected at different angles. As the water content of the aggregates increased, the presence of surface water film would cause changes in the reflected light. Therefore, the system could quantify the thickness of the water film by analyzing the reflected signal, and further judge whether the aggregates reached the SSD state. Figure 4b depicts the incidence and reflection of infrared light.

During *Infrared*, both the reflected signal and sample mass would be recorded. The former is used to judge the SSD state of the aggregates, while their W_SSD_ can be calculated using the latter. You et al. [71] compared *Infrared* with *Pycnometer*, and found that the results of *Infrared* have a smaller standard deviation, indicating that *Infrared* has a higher accuracy. In addition, the system can also be used to measure dry specific gravity, saturated-surface-dried specific gravity, and apparent specific gravity of fine aggregates [72].

However, although *Infrared* is more accurate since it reduces operation errors, it is still not widely popularized due to its high dependency on equipment. Furthermore, the existing water film coefficient formula is not suitable for RFA, because the characteristic wetting curve of RFA in the SSDetect system is unknown. Even so, *Infrared* is still a promising method to measure the W_SSD_ of RFA due to its high convenience and accuracy.

### 3.4. Evaporation

**Figure 5:**
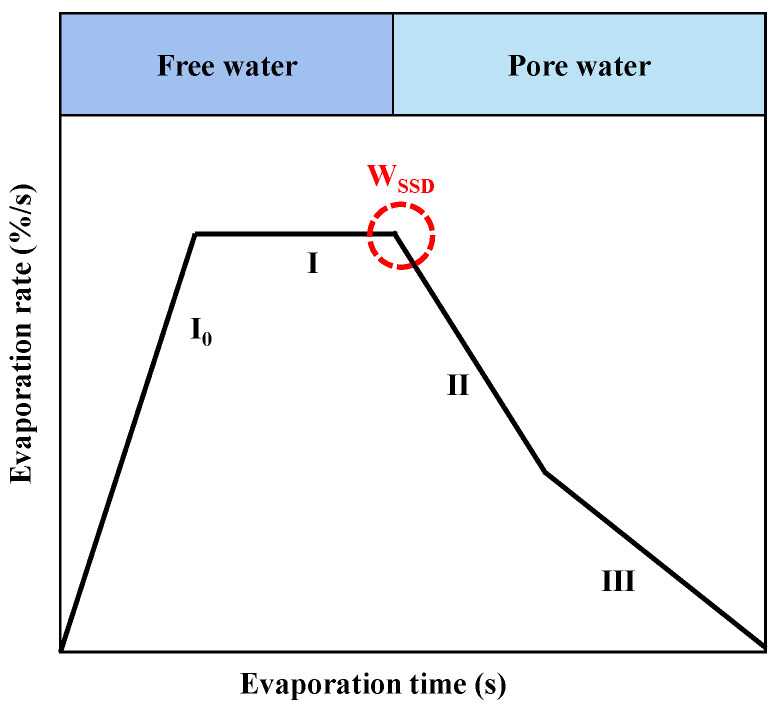
Relationship between the evaporation rate and the time. Note: stages I_0_, I, II, and III represent the increase of the water temperature from ambient one to evaporation one, the fast evaporation of free water, the water evaporation of large pores, and the water evaporation of small pores, respectively.

Mechling et al. [73] first proposed the relationship between the moisture state of fine aggregates and the water evaporation rate and further developed a new method for measuring the W_SSD_ of the aggregates. The authors [73] believed that the surface water of the aggregates would gradually escape under a constant high temperature, as shown in stages I_0_ and I in Figure 5; after the surface water evaporated completely, the pore water would begin to evaporate. Note that the evaporation rate of pore water would be slower than that of surface water due to capillary action and its small evaporation surface, as shown in stages II and III in Figure 5. Therefore, the W_SSD_ of the aggregates can be calculated using the mass at the inflection point between stages I and II.

Clearly, the drying temperature is the main factor during *Evaporation* [74]. A higher temperature may change the morphology and microstructure of fine aggregates, e.g., the dehydration of AFt in RFA [75,76], while a lower temperature can significantly prolong the test duration. However, there is no unified conclusion about the drying temperature. For example, Mechling et al. [73] used 40–50 °C to dry NA, while Damineli et al. [77] applied 70 °C for RFA. In addition, the influence of the ‘edge effect’ should be also considered. Based on a lot of experiments, we found that the water in the center of the drying plane always evaporated more slowly than that in the edge. Consequently, when the aggregates in the center reached the SSD state, the edge has been at the AD state.

### 3.5. Airflow Drying

**Figure 6:**
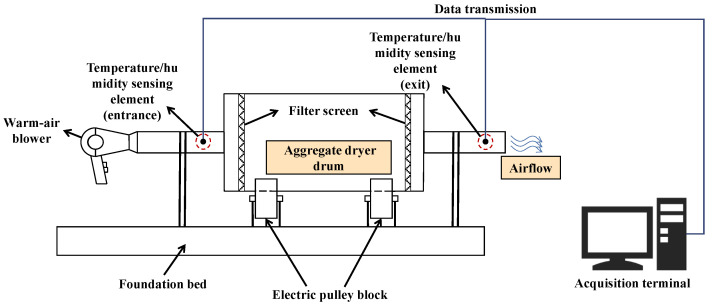
Schematic diagram of airflow drying equipment.

Similar to *Evaporation*, Kandhal et al. [78] first proposed a new method to measure the W_SSD_ of fine aggregates and the authors paid more attention to changes in temperature and relative humidity. As shown in Figure 6, the aggregates at the OS state are placed in a tumbling drum, a thermal airflow with constant temperature and humidity is continuously injected from the inlet, and a temperature-humidity-combined detector is installed at the outlet. When the surface water of the aggregates evaporates, the temperature of the airflow at the outlet drops, since its heat is absorbed. Therefore, there is a temperature gradient of the airflow from the inlet to the outlet. Correspondingly, the humidity of the airflow at the outlet surges due to the evaporation of surface water, resulting in a relative humidity gradient of the airflow from the inlet to the outlet.

**Figure 7:**
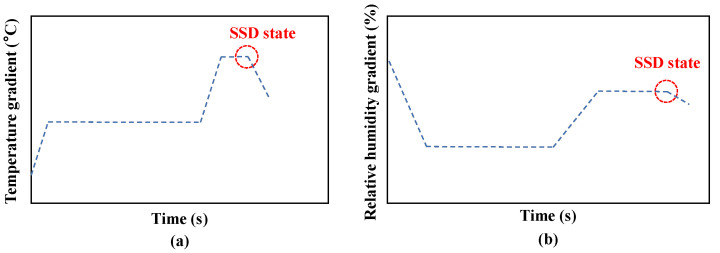
Judgment of the inflection point at which the temperature gradient (**a**) and relative humidity gradient (**b**) begin to decrease.

As the test progresses, the surface water of the aggregates totally evaporates. Similar to *Evaporation*, the evaporation rate of the pore water slows down. Therefore, the relative humidity gradient decreases. At the same time, because the mass of the pore water is less than the surface water, the heat absorbed by the pore water is less and the temperature gradient is also reduced. Gentilini et al. [79] defined the inflection point at which the temperature gradient and relative humidity gradient begin to decrease as the moment when the aggregates reach the SSD state, as shown in Figure 7.

Compared with the temperature gradient, Kandhal et al. [78] suggested that the relative humidity gradient could be regarded as a more effective indicator to measure the W_SSD_ of fine aggregates. Gentilini et al. [79] indicated that this conclusion could also be suitable for fillerised aggregates. This is because the temperature change has a longer delay than the humidity change and the heat transfer inside the equipment is always affected by many uncertainties.

Since the judgment of the inflection point depends on the variation trend rather than the specific value, the calculation errors of *Airflow drying* would be greatly reduced. However, relative humidity has a great relationship with temperature, i.e., an increase in temperature would lead to a decrease in relative humidity. It indicates that the delay and uncertainty of the temperature gradient would further negatively impact the relative humidity gradient. Therefore, the inflection point in the relative humidity curve always appears later than the moment when the aggregates reach the SSD state, resulting in the underestimation of W_SSD_.

### 3.6. Conductivity

**Figure 8:**
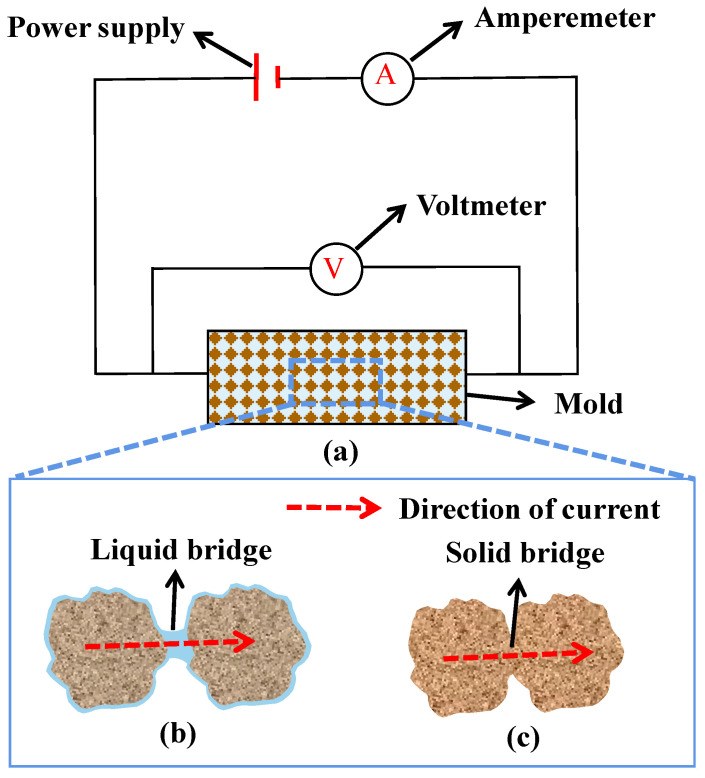
Schematic diagrams of the conductivity test (**a**), liquid bridges (**b**), and solid bridges (**c**).

Although the above methods have been proposed to measure the W_SSD_ of RFA, the errors of these methods are still large, especially for the W_SSD_ of finer RFA. This is because the content of impurities in RFA significantly increases with the decrease in particle size. Consequently, finer RFA is more like silt, is more prone to agglomerate, and encapsulates more water. For this purpose, *Conductivity* is proposed [63]. By measuring the conductivity of RFA compacted in the mold (Figure 8a), a curve between the water content and the conductivity of RFA can be established. It is found that when RFA is dried from the OS state to the AD state, there is always an obvious inflection point in the curve, regardless of particle size. Some possible explanations are listed as follows. On the one hand, when RFA is at the OS state, both the aggregate surface and the inter-particle voids are filled with water, which facilitates the flow of electric current. Therefore, a high conductivity can be detected. On the other hand, when RFA is at the AD state, the surface water disappears and the pores are not filled with water, resulting in a significant decrease in the conductivity. Consequently, an obvious change in conductivity can be found in the transition region between the OS state and the AD state, and Kim et al. [63] attributed this region to the SSD state of RFA.

Previous studies [80,81,82] also gave a similar explanation and introduced the concept of ‘bridges’. As shown in Figure 8b, ‘liquid bridges’ can be formed due to the presence of the surface water of RFA. In contrast, when the surface is dry, ‘liquid bridges’ would disappear. At this moment, the current can only pass through ‘solid bridges’ formed by the contact of aggregates with each other, as shown in Figure 8c. Consequently, the conductivity drops sharply. In general, the number of bridges is related to the content of the surface water and the number of inter-particle contacts, while the latter is usually regarded as a given value as the bulk density of RFA remains unchanged after being compacted. Therefore, the number of bridges mainly depends on the content of the surface water, while the former is reflected macroscopically as conductivity. As a result, the change in the content of the surface water of RFA can be well exhibited by the change of conductivity.

It should be noted that owing to different water contents, RFA with the same bulk density may have different numbers of inter-particle contacts. This is because when the water content is large enough, finer RFA may float in the inter-particle water, leading to the change in the number of inter-particle contacts. Furthermore, the curve between the conductivity and the water content of RFA usually requires repeated measurements to be verified and calibrated and each measurement includes multiple drying-absorbing processes. Therefore, *Conductivity* would incur a significant amount of time and costs.

Even so, *Conductivity* still has high feasibility. Sosa et al. [80] investigated a ternary relationship between the water content, the conductivity, and the slumped shape of RFA. It can be clearly seen that when RFA is prepared based on the W_SSD_ calculated by *Conductivity*, its slumped shape perfectly conforms to the requirements of ASTM C128-12 [61]. Therefore, for finer RFA with a particle size of < 2 mm, judging its SSD state by *Conductivity* may be a promising method.

### 3.7. Pycnometer

Compared with other methods, *Pycnometer* has a low dependence on equipment and only needs a glass-type graduated pycnometer and an electronic balance. Based on absorption kinetics, the operation steps of *Pycnometer* are listed as follows [57]: (1) placing dried RFA of which the mass is recorded as M_dry_ into the bottle and adding a little water; (2) stopping adding water and gently shaking the bottle to discharge bubbles from the pores and the voids between particles; (3) adding water again until the liquid level reaches a certain height and recording the weight as $G_{\mathrm{total}}^{\mathrm{begin}}$, including the bottle, water, and RFA; (4) after immersion for a certain time, adding water again until the liquid level reaches the initial height because the liquid height drops after RFA absorbs water; (5) gently shaking the bottle and recording the weight; and (6) repeating steps (4) and (5) until the liquid level no longer drops and recording the weight as $G_{\mathrm{total}}^{\mathrm{end}}$. The W_SSD_ of RFA can be calculated by Equation (5).

(5) $$W_{\mathrm{SSD}}=\frac{G_{\mathrm{total}}^{\mathrm{end}}{-G}_{\mathrm{total}}^{\mathrm{begin}}}{M_{\mathrm{dry}}}\times 100\%$$

Obviously, the premise of *Pycnometer* is that RFA should be kept at the OD state during the first addition of water and it should start to absorb water when the liquid level first reaches the initial height. In other words, during the calculation for $G_{\mathrm{total}}^{\mathrm{begin}}$, the absorption amount of RFA should be zero. However, in fact, a part of the water can be absorbed by RA at the OD state in only several seconds during the addition of water [83], which would lead to the overestimation of $G_{\mathrm{total}}^{\mathrm{begin}}$ and the underestimation of W_SSD_. Castro et al. [57] reported a similar phenomenon. Furthermore, impurities and bubbles in RFA may float on the liquid level, making it difficult to judge the position of the latter. Therefore, the results of *Pycnometer* strongly depend on the operator’s experience.

To reduce the pre-absorption of RFA before the calculation for $G_{\mathrm{total}}^{\mathrm{begin}}$, one of the promising methods is the addition of sodium hexametaphosphate (SHMP), a clean, non-toxic phosphate that is widely used as clay-dispersing agent [84,85]. Previous studies [44,86] reported that the discharge rate of bubbles in RFA was significantly faster in the bottle adding SHMP, compared with the one without SHMP. It confirms that SHMP can help to discharge inter-particle air bubbles and shorten the time taken in step (2). Its effect would be more significant for finer RFA that is prone to be agglomerated due to a high content of impurities.

### 3.8. Hydrostatic Balance

**Figure 9:**
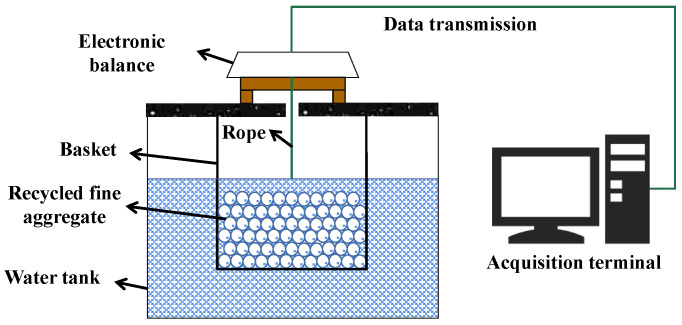
Schematic diagram of the hydrostatic balance test.

Except for magnitude, the time-dependent law of the W_a_ of RFA has also attracted huge attention. For this purpose, *Hydrostatic balance* has been developed based on Archimedes’ principle and absorption kinetics. As shown in Figure 9, *Hydrostatic balance* involves an electronic balance, a water tank, a mesh basket with holes, and data transmission and collection equipment connected with the balance. Here, the balance can measure the mass change of the basket and RFA immersed in the tank.

Before adding RFA, the reading of the balance should be reset to zero. Then, RFA at the OD state is put lightly into the basket. At this moment, three forces are acting on RFA, including gravity ($G_{\mathrm{RA}}^{0}$), the support force of the mesh basket ($F_{a}^{0}$), and buoyancy ($F_{b}^{0}$), while a force (i.e., the pull force from the basket, $G^{0}$) is acting on the balance. Here, $G^{0}$ is equal to $F_{a}^{0}$. Based on force analyses, Equation (6) can be obtained. As the test progresses, the pores of RFA are gradually filled with water, while bubbles in the pores are correspondingly discharged. At any time, the gravity of RFA, the support force of the basket, the buoyancy of RFA, and the pull force from the basket are recorded as $G_{\mathrm{RA}}^{t}$, $F_{a}^{t}$, $F_{b}^{t}$, and $G^{t}$, respectively. Since the volume of discharged bubbles in the pores is the same as that of absorbed pore water, the buoyancy of RFA remains unchanged. Here, $F_{b}^{0}$ is equal to $F_{b}^{t}$. Therefore, Equation (7) and Equation (8) can be obtained and the $W_{a}^{t}$ of RFA can be calculated by Equation (8).

(6) $$G_{\mathrm{RA}}^{0}{=F}_{a}^{0}{+F}_{b}^{0}{=G}^{0}{+F}_{b}^{0}$$

(7) $$G_{\mathrm{RA}}^{t}{=F}_{a}^{t}{+F}_{b}^{t}{=F}_{a}^{t}{+F}_{b}^{0}{=G}^{t}{+F}_{b}^{t}{=G}^{t}{+F}_{b}^{0}$$

(8) $$\Delta G_{\mathrm{RA}}=\Delta F_{a}=\Delta G$$

An advantage of *Hydrostatic balance* is that it avoids the step of judging the SSD state of RFA. Furthermore, because of its high operability and simple equipment, it has been highly recommended by many scholars. Tam et al. [87] investigated the W_SSD_ of RFAs with different sources and found that the standard deviation of all results was only around 1%, indicating a high accuracy of *Hydrostatic balance*. Other studies [83,88,89,90] further reported that the $W_{a}^{t}$ of RFA immersed for 24 h could be regarded as its W_SSD_.

Note that the absorption amount of RFA in the first few seconds is extremely high, as mentioned in Section 2.1. According to Tegguer et al. [46], within the first few seconds, the absorption rate of RFA can reach the peak value and the absorption amount can also reach more than 25% of its total absorption. However, at the beginning of *Hydrostatic balance*, since the addition of RFA disturbs the water surface and the basket, the absorption amount of RFA in the first few seconds is usually neglected. Consequently, Rodrigues et al. [86] indicated that the W_SSD_ of RFA measured by *Hydrostatic balance* was always smaller than that measured by *Pycnometer*. In addition, the general applicability of *Hydrostatic balance* needs to be further investigated, because finer RFA with a particle size of < 1.18 mm may float in water, resulting in a smaller value of $G^{0}$ and a larger value of W_SSD_.

### 3.9. Extrapolation

Considering that existing methods usually lead to inaccurate results for the W_SSD_ of finer RFA, Zhao et al. [91] proposed a new method with two assumptions, namely *Extrapolation*. On the one hand, the W_a_ of RFA is equal to the sum of the W_a_ of OM ($W_{a}^{\mathrm{OM}}$) and the W_a_ of NA ($W_{a}^{\mathrm{NA}}$). On the other hand, the $W_{a}^{\mathrm{OM}}$ does not vary with the particle size of RFA or OM. More specifically, the W_SSD_ and the mass ratio of OM ($M_{\mathrm{OM}}$) of coarser RFA should be first determined by *Slumping* and the dissolution method using salicylic acid, respectively. Then, Equation (9) can be established by linearly fitting the $M_{\mathrm{OM}}$ and the W_SSD_ of coarser RFA. After that, Equation (9) is directly applied to finer RFA. Therefore, the W_SSD_ of finer RFA can be calculated using its $M_{\mathrm{OM}}$.

(9) $$W_{\mathrm{SSD}}{=W}_{a}^{\mathrm{OM}}{\times M}_{\mathrm{OM}}{+W}_{a}^{\mathrm{NA}}{\times (1-M}_{\mathrm{OM}})$$

Le et al. [92] further assumed that the $M_{\mathrm{OM}}$ was proportional to the mass loss of RFA at 475 °C (ML_475_), and optimized the calculation process of the W_SSD_ of RFA, as shown in Equation (10) and Equation (11). Furthermore, Delobel et al. [21] suggested that because the $W_{a}^{\mathrm{NA}}$ was so small that it could be ignored, the calculation of the W_SSD_ of RFA could be further optimized, as shown in Equation (12).

(10) $$M_{\mathrm{OM}}{=k\times \mathrm{ML}}_{475}$$

(11) $$W_{\mathrm{SSD}}{=(W}_{a}^{\mathrm{OM}}{-W}_{a}^{\mathrm{NA}}{)\times k\times \mathrm{ML}}_{475}{+W}_{a}^{\mathrm{NA}}$$

(12) $$W_{\mathrm{SSD}}{=W}_{a}^{\mathrm{OM}}{\times k\times \mathrm{ML}}_{475}$$

According to Zhao et al. [91], when the PC is given, the porosity of OM in RFA does not change, and thus the $W_{a}^{\mathrm{OM}}$ of RFA with different particle sizes is also unchanged. However, in fact, the smaller the particle size of RFA, the higher the crushing degree, and the more cracks and pores in OM [93]. Furthermore, with the decrease in particle size, the specific surface area of RFA would significantly increase [64]. Therefore, more attempts should be made to improve the assumptions of *Extrapolation*. Even so, its feasibility has been confirmed in several studies [91,94]. In particular, compared with existing methods, it has more potential to measure the W_SSD_ of RFA, because it has higher repeatability and a low dependence on an operator’s experience.

## 4. Prospects

The details of existing methods for measuring the W_SSD_ of RA have been discussed above. It can be clearly seen that although these methods are feasible to some degree, the accuracy of all test results is not satisfactory due to the flaws in physical principle or operability. Table 4 summarizes the working scenarios, advantages, and disadvantages of these methods. Obviously, it is crucial to provide some suggestions to develop an accurate and convenient method for measuring the W_SSD_ of RA.

### 4.1. Suggestions for Slumping

It was found that the W_SSD_ of RFA was always larger than RCA and the measurement of the former was more difficult. Therefore, we paid more attention to the optimization of *Slumping*. As shown in Section 3.1.2, when the mold is lifting, there are at least three forces acting on RFA, including friction, the surface tension of water, and the aggregate interlocking. At this time, the force analysis of RFA is so complicated that the physical principle of *Slumping* is still unclear. In particular, the characteristics (i.e., magnitude, direction, and action spot) of these forces may vary with the change in particle size. Furthermore, the inter-particle water of agglomerate impurities in finer RFA also needs to be considered. Therefore, more attempts should be made to elucidate the physical principle of *Slumping* and its relationship to the size of RFA. Note that the definition of the slumped shapes should be more explicit to avoid the interference of human factors.

### 4.2. Suggestions for Heat Transfer

As shown in Section 3.4 and Section 3.5, *Evaporation* and *Airflow drying* are feasible in theory because their physical principles are sound. However, the operability of *Evaporation* and *Airflow drying* is not satisfactory. For example, there are always samples on the center and edge of the drying plane during *Evaporation*, leading to the inevitable ‘edge effect’. In addition, the inflection point in *Airflow drying* always appears later than the moment representing RFA at the SSD state due to the delay in changes to the temperature gradient and the relative humidity gradient.

Here, we suggest combining *Evaporation* and *Airflow drying* because the mass change usually has no delay and the ‘edge effect’ is absent in a tumbling drum. More specifically, RFA at the OS state can be placed in a tumbling drum, a thermal airflow with constant temperature and humidity can be continuously injected from the inlet, and a mass detector can be installed at one side of the drum. Then, for each tumble, the mass of RFA can be recorded until an inflection point in the curve between the mass of RFA and the tumbling number appears. Furthermore, there is a promising solution to overcome the situation where an accurate inflection point may be missed because the curve belongs to the scatter plot, leading to the calculation errors of W_SSD_. We suggest conducting the tumble with multiple rounds: (1) determining the approximate range of the inflection point for the first round; (2) shortening the range of the inflection point by increasing the angular velocity of the drum for the second round; and (3) repeating step (2) until the error of the inflection point is within the acceptable range.

Finally, it has been widely accepted that determining an appropriate temperature is crucial for heat transfer processes. However, according to Théréné et al. [38], if the W_a_ of RA is measured with the same preparation protocol, e.g., the same drying temperature, the correction is not needed and the effect of drying temperatures on the W_SSD_ of RA is also negligible.

### 4.3. Suggestions for Absorption Kinetics

Similar to *Evaporation* and *Airflow drying*, the physical principles of *Pycnometer* and *Hydrostatic balance* are sound, but their operability is relatively poor. In particular, compared with *Pycnometer*, *Hydrostatic balance* seems more advanced because it has been widely used to investigate the time-dependent law of W_a_. Therefore, we would like to provide suggestions for *Hydrostatic balance*.

As shown in Section 3.8, there are two major problems with *Hydrostatic balance*. On the one hand, finer RFA with a particle size of < 1.18 mm would float in water. To solve this problem, it is crucial to make the gravity of finer RFA larger than its buoyancy. According to Archimedes’ principle, the most effective way is to lower the density of a liquid to less than that of the finer RFA, because the volume of the latter has been small enough. In other words, the tap water should be replaced by a liquid with a lower density. On the other hand, RFA has absorbed a part of water before determining the initial reading, leading to the underestimation of its W_SSD_. One of the promising methods is the use of a liquid of which the viscosity can significantly change with temperature and/or pressure. In the beginning, when the viscosity is large enough, the absorption amount of RFA can be neglected before determining the initial reading. After that, we can control the absorption rate of RFA by adjusting the temperature and/or pressure upon the liquid.

Based on the above analysis, in *Hydrostatic balance*, we suggest using a liquid with a lower density and of which the viscosity can significantly change with temperature and/or pressure instead of water. Kerosene or paraffin may be a feasible choice. Justin Sundararaj et al. [95] indicated that with the increase in the temperature from 26 to 60 °C, the viscosity of modified kerosene with the density of approximately 0.80 g/cc decreased from 1.33 to 0.90 cP.

### 4.4. Suggestions for Conductivity

As mentioned in Section 3.6, it is assumed in *Conductivity* that the number of inter-particle contacts is always a given value after RFA has been compacted in the mold. However, because finer RFA may float in the inter-particle water, RFA with the same bulk density may have different numbers of inter-particle contacts. Therefore, more attempts should be made to investigate the effects of water content on the bulk density of RFA and the number of inter-particle contacts.

### 4.5. Suggestions for other Methods

It can be obviously observed that the applications of *Infrared* and *Extrapolation* usually need specific conditions, such as the equipment named the SSDetect system. Therefore, suggestions about *Infrared* and *Extrapolation* are reserved in this review.

In contrast, *Centrifugation* is a relatively mature method to measure the W_SSD_ of RA because of its sound physical principle that provides a way to distinguish the pores with and without water by adjusting the rotation speed of the centrifuge. However, *Centrifugation* is usually accompanied by a prominent problem. Since the open-ended direction of pores in OM is random while the direction of centrifugal force is fixed, it is difficult to dry all large pores and saturate all small pores. Here, we suggest adding a subminiature mixer to each centrifuge tube and simultaneously stirring in the tubes during *Centrifugation*.

## 5. Conclusions

In this review, an overview of existing methods for measuring the W_SSD_ of RA is presented, including *Wiping*, *Slumping*, *Centrifugation*, *Infrared*, *Evaporation*, *Airflow drying*, *Conductivity*, *Pycnometer*, *Hydrostatic balance*, and *Extrapolation*. The physical principles and operability of these methods are mainly discussed. Several improvement suggestions are also provided. In addition, the absorption behaviors and moisture states of RA are also described. The main results are shown as follows:

(1) Compared with NA, the absorption behaviors of RA have four outstanding features, including higher capacity, larger standard deviations, and time and size dependency. Furthermore, RA has five categories of moisture states. The AD state is an ideal moisture state for RA and the SSD state is usually used to evaluate the additional water of RAC.
(2) In most standards, *Wiping* and *Slumping* are used to measure the W_SSD_ of RCA and RFA, respectively. However, the operation of *Slumping* heavily depends on the operator’s experience because of vague definitions of the slumped shape of RFA. Furthermore, the physical principle of *Slumping* and its relationship to particle size are still unclear.
(3) The physical principles of *Evaporation*, *Airflow drying*, *Pycnometer*, *Hydrostatic balance*, and *Centrifugation* are almost faultless. However, many problems exist in the operation of these methods. For example, the water on one side of RA is largely retained during *Centrifugation*. Furthermore, during *Hydrostatic balance*, RFA would absorb a part of water before determining the initial reading.
(4) Although *Infrared*, *Conductivity*, and *Extrapolation* are easy to operate, their physical principles need to be improved. For instance, it is crucial for *Infrared* to update the water film coefficient formula to be suitable for RFA. In addition, the influence of water content on the bulk density of RFA and the number of inter-particle contacts in *Conductivity* should be clarified.
(5) To develop an accurate and convenient method for measuring the W_SSD_ of RA, several suggestions are proposed, including the combination of *Evaporation* and *Airflow drying* where the mass change of RFA can be recorded after each tumble, the liquid selection in *Hydrostatic balance* (e.g., kerosene or paraffin), and the addition of a tiny mixer in each centrifuge tube.

## Figures and Tables

**Table 1:** Details of five moisture states of RA.

Moisture States	Schematic Diagram	Description
Oven dry (OD)	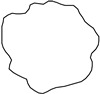	The pores inside the aggregate are completely dry, and there is no water on the surface.
Air dry (AD)	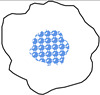	The bottom of the pores inside the aggregate is filled with water, while the top of the pores near the surface keeps dry [46]. Meanwhile, there is no water on the surface.
Surface saturation surface-dry (SSSD)	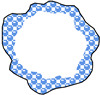	The bottom of the pores inside the aggregate keeps dry, while the top of the pores near the surface is filled with water [46]. Meanwhile, there is no water on the surface. It generally happens on the pores with a large aspect ratio.
Saturated surface-dry (SSD)	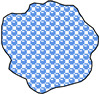	The pores inside the aggregate are filled with water, while there is no water on the surface.
Over saturation (OS)	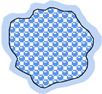	The pores inside the aggregate are filled with water, and there is a water film on the surface.

**Table 2:** Details of *Wiping* in different reports.

Reference	Tool	Procedure	Judgment
NF EN 1097-6 [53]	Dry towel	(1) Transferring aggregates to a dry towel; (2) Removing the surface water by gently rolling them in the towel for not more than 15s.	All visible films of water are removed, but aggregates still have a damp appearance.
BS 812: part 2 [54]			
ASTM C127-12 [55]		(1) Removing aggregates from the water; (2) Rolling them in a large absorbent towel; (3) Wiping larger particles individually.	
GB/T 25177 [56]		(1) Holding both ends of the towel to make it into a groove shape; (2) Letting aggregates roll back and forth on the towel 8 to 10 times.	-
Castro et al. [57]	Dry tissue	Patting the surface of aggregates dry with a dry tissue.	Visual inspection: It appears that the tissue is no longer absorbing moisture from aggregates.
IFSTTAR. Test Method No. 78 [59]	Colorful absorbent tissue	Drying aggregates progressively with different sheets of colorful absorbent tissues.	No trace of water can be seen on the tissues.

**Table 3:** Details of *Slumping* in different reports.

Standards	Procedures	Judgment
ASTM C128-12 [61]	(1) Filling aggregates loosely in the mold; (2) Tamping aggregates into the mold with 25 light drops; (3) Starting each drop approximately 5 mm above the top surface of aggregates; (4) Permitting the tamper to fall freely under gravity on each drop; (5) Adjusting the starting height to the new surface elevation after each drop; (6) Removing excess aggregates from the base; (7) Lifting the mold vertically.	Slight slumping of the molded fine aggregate indicates that it has reached a surface-dry condition.
AASHTO T-84 [62]	(1) Filling the mold to overflowing with aggregates; (2) Lightly tamping aggregates into the mold with 25 light drops; (3) Starting each drop about 1/5 in. above the top surface of aggregates; (4) Removing excess aggregates from the base; (5) Carefully lifting the mold vertically.	When the fine aggregate achieves an SSD condition, the fine aggregate will slump.
EN 1097-6 [53]	(1) Filling the mold loosely with aggregates; (2) Placing the metal tamper on the surface of aggregates through the hole at the top of the mold; (3) Tamping the surface 25 times by letting the tamper fall under its own weight; (4) Gently lifting the mold clear of aggregates.	The collapse situation occurs at mold removal.
GB/T 14684 [60]	(1) Placing aggregates into the mold in two layers, where the first layer is half of the mold height; (2) Evenly tamping it 13 times with a tamper from about 10mm from the surface of aggregates under gravity; (3) Filling the second layer into the mold; (4) Repeating step 2; (5) Lifting the mold vertically and slowly after the upper mouth of the mold is flattened.	Repeating the operation until fine aggregates reach a fixed shape.

**Table 4:** Working scenarios, advantages, and disadvantages of existing methods.

Methods	Scenarios	Advantages	Disadvantages
*Wiping*	Laboratory and construction site	(1) Simple operation; (2) Low dependence on equipment; (3) Time-saving.	(1) High dependency on operators’ experience; (2) Loss of adhered OM due to the squash of towels/tissues.
*Slumping*			(1) Unclear physical principles; (2) Large error when applied to finer RFA; (3) High dependency on an operator’s experience; (4) Vague definition of the slumped shapes.
*Centrifugation*	Laboratory	(1) Simple operation; (2) Low dependence on operators’ experience; (3) Time-saving; (4) Customizable maximum pores containing water.	(1) Conflicting relationship between the random open-ended direction of pores and a fixed direction of centrifugal force; (2) High dependence on equipment.
*Infrared*		(1) Simple operation; (2) Low dependence on operators’ experience; (3) Time-saving; (4) Available other data (e.g., specific gravity).	(1) Unsuitable water film coefficient formula for RFA; (2) High dependence on equipment.
*Evaporation*		(1) Simple operation; (2) Low dependence on equipment; (3) Low dependence on operators’ experience.	(1) Unknown drying temperature; (2) Edge effect; (3) Time-consuming.
*Airflow drying*		(1) Simple operation; (2) Low dependence on operators’ experience.	(1) Delay of changes in the temperature and relative humidity gradients; (2) High dependence on equipment; (3) Time-consuming.
*Conductivity*	Laboratory and construction site	(1) Simple operation; (2) Low dependence on equipment; (3) Low dependence on operators’ experience.	(1) Unclear effects of water content on the bulk density of RFA and the number of inter-particle contacts; (2) Time-consuming.
*Pycnometer*		(1) Simple operation; (2) Low dependence on equipment; (3) Time-saving.	(1) High dependency on an operator’s experience; (2) Pre-absorption of RA before determining the initial reading; (3) Difficult judgment of the position of the liquid level.
*Hydrostatic balance*	Laboratory	(1) Simple operation; (2) Low dependence on equipment; (3) Low dependency on operators’ experience; (4) Time-saving; (5) Available time-varying law.	(1) Pre-absorption of RA before determining the initial reading; (2) Floating finer RFA.
*Extrapolation*		(1) Not affected by RFA sizes; (2) Low dependency on operators’ experience.	(1) Incomplete assumptions; (2) Time-consuming; (3) Complicated operation.
